# A conserved aspartate residue in [4Fe-4S]-containing HypD is required for [NiFe]-cofactor biosynthesis and for efficient interaction of the HypCD scaffold complex with HypE

**DOI:** 10.1093/mtomcs/mfaf014

**Published:** 2025-05-24

**Authors:** Alexander Haase, Christian Arlt, Maximilian Hardelt, Andrea Sinz, R Gary Sawers

**Affiliations:** Institute for Biology/ Microbiology, Martin Luther University Halle-Wittenberg, Halle (Saale), Germany; Institute of Pharmacy, Center for Structural Mass Spectrometry, Martin Luther University Halle-Wittenberg, Halle (Saale), Germany; Institute for Biology/ Microbiology, Martin Luther University Halle-Wittenberg, Halle (Saale), Germany; Institute of Pharmacy, Center for Structural Mass Spectrometry, Martin Luther University Halle-Wittenberg, Halle (Saale), Germany; Institute for Biology/ Microbiology, Martin Luther University Halle-Wittenberg, Halle (Saale), Germany

**Keywords:** cyanide ligand, [Fe-S]-protein HypD, hydrogenase, Hyp proteins, maturation, [NiFe]-cofactor

## Abstract

Six Hyp (A through F) proteins synthesize the NiFe(CN)_2_CO cofactor found in all [NiFe]-hydrogenases. The Fe(CN)_2_CO moiety of this cofactor is assembled on a separate scaffold complex comprising HypC and HypD. HypE and HypF generate the cyanide ligands from carbamoyl phosphate by converting the carbamoyl moiety to a thiocyanate associated with HypE’s *C*-terminal cysteine residue, within a conserved ‘PRIC’ motif. Here, we identify amino acid residue D98 in the central cleft of HypD to be required for biosynthesis of the Fe(CN)_2_CO moiety and for optimal interaction of HypD with HypE. Construction of a D98A amino acid variant of HypD caused near-complete loss of hydrogenase activity in anaerobically grown *Escherichia coli* cells, while exchange of the structurally proximal, but non-conserved, residue S356 on HypD, did not. Native mass spectrometric analysis of the anaerobically purified HypC-HypD_D98A_ scaffold complex revealed only a low amount of the bound Fe(CN)_2_CO group. Western blotting experiments revealed that purified scaffold complexes between either HypC or HybG (a paralogue of HypC) with HypD-D98A showed a strongly impaired interaction with HypE. Examination of the HypCDE complex crystal structure from *Thermococcus kodakarensis* revealed that D98 of HypD lies within a cleft through which the *C*-terminus of HypE can access the bound iron ion on HypCD. Alphafold3 predictions suggest that the D98 residue interacts with the arginine residue of the ‘PRIC’ motif at the *C*-terminus of HypE to position the modified terminal cysteine residue precisely for delivery of cyanide to the iron ion associated with the HypCD complex.

## Graphical Abstract

**Graphical Abstract:**
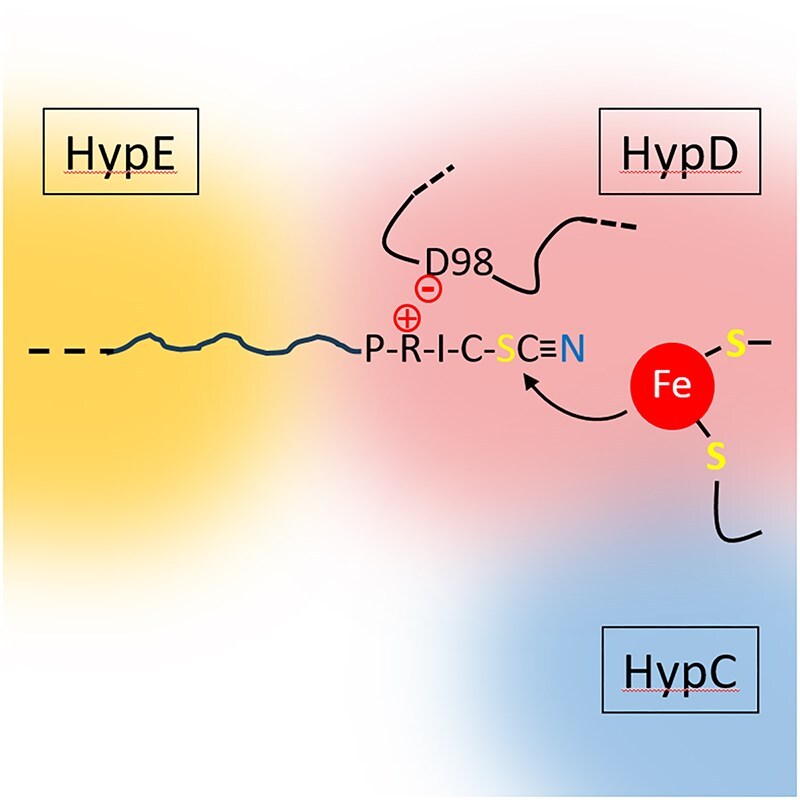
The *C-*terminal cysteine residue of HypE is on a flexible peptide sequence bearing a highly conserved ‚PRIC’ motif. Postioning of the thiocyanate modification on the cysteine close to the Fe ion coordinated by the HypCD complex is facilitated by an electrostatic interaction between the D98-carboxylate of HypD and R334 of HypE’s ‚PRIC‘ motif.

## Introduction

The biosynthesis of the bimetallic NiFe(CN)_2_CO cofactor found in the catalytic subunit of all [NiFe]-hydrogenases (Hyd) requires the involvement of six conserved Hyp (hydrogenase pleiotropy) proteins [1–3]. The cofactor is synthesized in four stages. Initially, the Fe(CN)_2_CO moiety of the cofactor is assembled on a scaffold complex comprising the HypC and HypD proteins. Biosynthesis of this Fe-moiety requires the activities of two further Hyp enzymes, HypE and HypF, which are necessary for synthesis and delivery of the two cyanide ligands; the metabolic origin of the CO ligand in anaerobic Hyds is currently unresolved and may require a further enzyme(s). The second stage involves the transfer of the Fe(CN)_2_CO moiety into the apo-catalytic subunit [1, 4]. Once this has occurred, then the nickel ion is introduced in a third stage by the combined actions of the HypA and HypB proteins [1–3, 5, 6]. The fourth and final stage of cofactor assembly in most, but not all [7], [NiFe]-hydrogenases involves the specific proteolytic removal of a *C*-terminal peptide from the catalytic subunit [1, 8], which causes a conformational change resulting in closure of the active site. This conformational change allows the subsequent interaction of the now holo-catalytic subunit with the holo-form of the iron–sulfur cluster-containing electron-transfer subunit to form the active enzyme complex [1, 9, 10].

Our current understanding of the synthesis of the cyanide ligands was advanced significantly by studying the three anaerobically synthesized [NiFe]-hydrogenases of *Escherichia coli* [11–13]. While Hyd-1 and Hyd-2 are mainly functional as H_2_-oxidising enzymes [14], Hyd-3 is exclusively associated with the formate hydrogenlyase (FHL-1) complex, which disproportionates formate into H_2_ and CO_2_ [15]. By using the activities of these enzymes as a test-bed, the Böck research group initially identified carbamoyl phosphate (CP) as the source of the cyanide ligands [16, 17]. However, initial hints as to CP being the source of the cyanides were derived from an earlier study of *Salmonella enterica* Typhimurium mutants lacking hydrogenase activity [18]. Together, these studies ultimately led to the elucidation of how HypF catalyses the adenosine triphosphate (ATP)-dependent transfer of the carbamoyl moiety of CP to the thiol group of the *C*-terminal cysteine residue on HypE [11, 12]. The resulting enzyme-bound thiocarbamate formed by this transferase reaction is then subsequently dehydrated to the thiocyanate by the ATP-dependent activity of HypE [11, 19].

One key feature of the *C*-terminus of HypE is that secondary structural predictions indicate that it has a highly flexible ‘tail’ [3], or ‘finger-like’, structure, which is borne out by the lack of a clearly defined structure for the *C*-terminus of HypE [3, 20]. The second key feature of the *C*-terminus of HypE is the presence of a highly conserved PRIC motif, common to all HypE proteins [1, 3, 11, 12]. This motif was shown to be essential for delivery of the cyanide moiety to the Fe ion associated with the HypCD scaffold [12], which specifically matures Hyd-3 in *E. coli*, and additionally to the Fe ion bound to the Hyd-2-specific HybG-HypD scaffold maturation complex [21].

**Figure 1.**
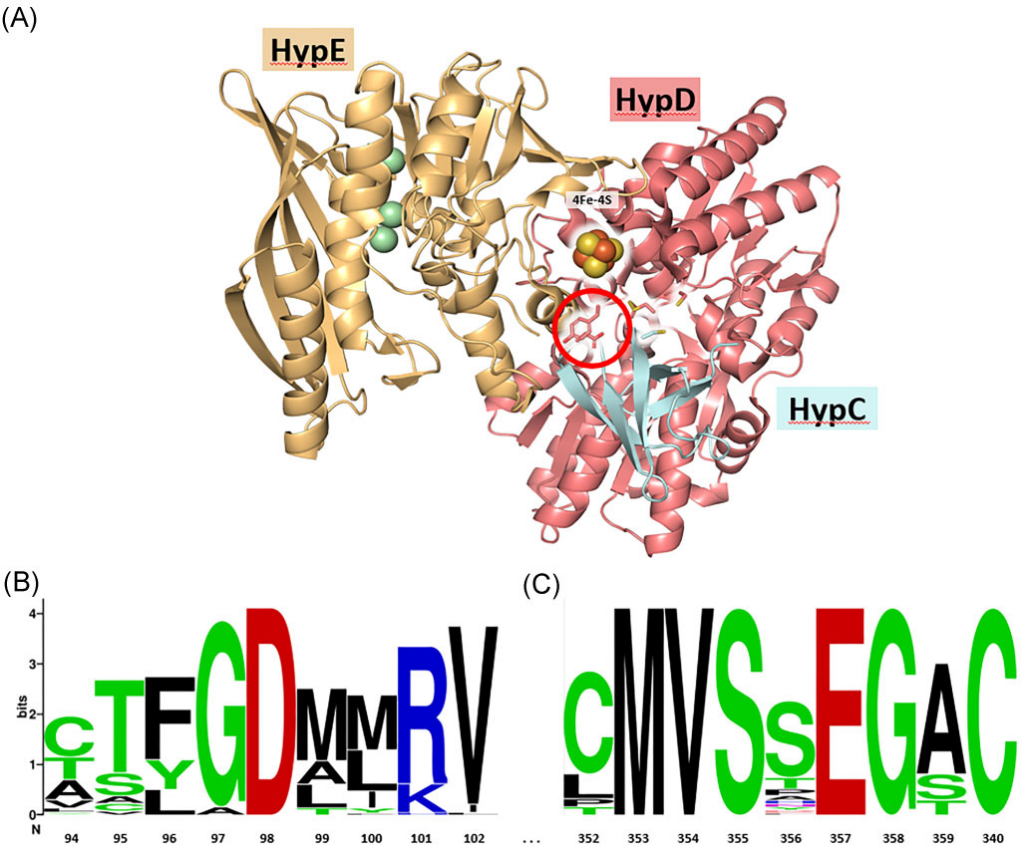
Location and conservation of D98 in *E. coli* HypD. (a) Structural representation of the HypCDE complex from *T. kodakarensis* (PDB: 3VYS) as determined by Watanabe et al. [20] and showing the locations of residues D100 and Y358 (equivalent to D98 and S356, respectively, in *E. coli* HypD), which are within the circle. The [4Fe-4S]-cluster in HypD is shown and the conserved cysteine residues in HypC, HypD, and HypE are highlighted. The spheres in HypE represent Mg^2+^ ions. (b) Amino acid alignment of a total of 70 HypD proteins, selected from representative archaea and bacteria (see Table S1), showing the residues in the immediate neighbourhood of D98, and, in part (c), in the immediate neighborhood of S356 in *E. coli* HypD.

Examination of the crystal structure of the HypCDE complex from *Thermococcus kodakarensis* [20] reveals that dimeric HypE associates transiently and side-on with the HypCD heterodimer (see Fig. 1a) and must ‘thread’ its *C*-terminus through a central cleft in HypD to deliver the cyanide ligand to the bound Fe ion [3], which is predicted to be coordinated by two highly conserved and essential cysteine residues, C41 on HypD and C2 on HypC and its paralogues [1, 13, 22].

Like HypE, HypD also bears many highly conserved motifs that are important for its function [23]. However, closer examination of the central cleft in HypD (depicted in Fig. 1a), through which HypE’s *C*-terminal tail is proposed to be threaded [3], reveals a highly conserved aspartic acid residue, D98, in *E. coli* numbering (Fig. 1b). In this study, we provide evidence supporting a role for this residue is aiding delivery of the cyanide moiety from dimeric HypE to the HypCD scaffold complex.

## Results

### Benzyl viologen (BV)-linked total hydrogenase enzyme activity is absent in a strain synthesising the D98A amino acid-exchange variant of HypD

Examination of the primary structure of HypD identified an almost universally conserved aspartic acid residue (D98) (Fig. 1b), which has not been previously considered as a target in amino acid-exchange programs. Based on the crystal structure of the HypCDE complex from *T. kodakarensis* [3, 20], D98 (*E. coli* numbering) is located within a cleft in HypD (Fig. 1a) that is conceivably involved in facilitating access of the highly flexible *C*-terminal PRIC-containing tail of HypE [1, 11] to deliver the cyanide ligands during biosynthesis of the Fe(CN)_2_CO moiety on the HypCD scaffold complex. To determine whether this conserved residue might be important for this proposed function, a D98A amino acid-exchange was introduced into HypD (see Experimental). As a control, a S356A residue-exchange in HypD of *E. coli* was undertaken, as it is proximal to D98 in the cleft in HypD (see below). This residue is not well conserved in HypD proteins (Fig. 1c), and indeed is replaced by a tyrosine in HypD from *T. kodakarensis* (Fig. 1a), and was chosen because it is not anticipated to impact maturation function.

**Figure 2.**
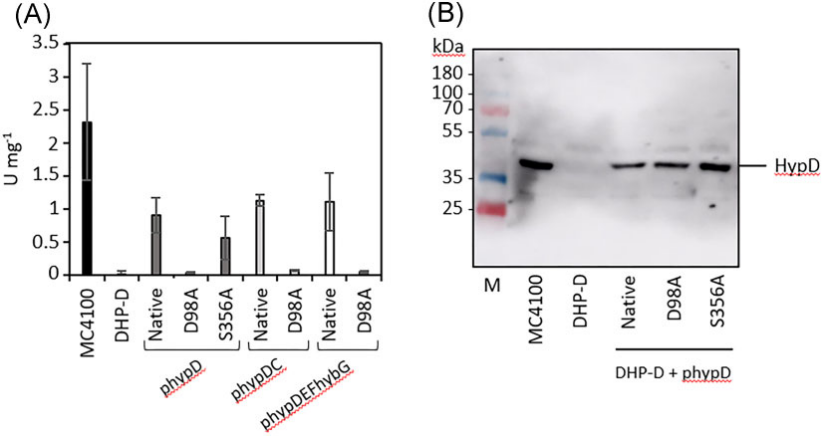
An *E. coli* strain synthesising HypD_D98A_ is severely impaired in H_2_: BV oxidoreductase enzyme activity. (a) Total hydrogenase (H_2_: BV oxidoreductase) specific activity was determined in cell-free extracts derived from the strains, MC4100 (parental WT), DHP-D (Δ*hypD*), and DHP-D transformed with either phypD, phypDC, or phypDEFhybG after anaerobic growth in TGYEP medium. Native indicates synthesis of native HypD; D98A indicates synthesis of HypD_D98A_; and S356A indicates synthesis of HypD_S356A_. Measurement of enzyme activity was performed with three biological and three technical replicates and data are presented together with standard deviation. (b) Immunoblot analysis of the same cell-free extracts (25 µg of protein) as used in part A and treated with anti-HypD antiserum (diluted 1:1000 v/v). Polypeptides in the extracts were separated in sodium dodecyl sulphate polyacrylamide gel electrophoresis (SDS-PAGE) (12.5% w/v acrylamide). The migration position of HypD is indicated on the right of the panel and migration positions of molecular mass markers (in kDa) are shown on the left. A representative blot is shown and the experiment was done twice.

To determine if and how these introduced amino acid exchanges in HypD affected the ability of the protein to support maturation of the three hydrogenases synthesized during fermentative growth of *E. coli*, different plasmids carrying the native or the modified versions of the *hypD* gene were introduced into *E. coli* strain DHP-D (Δ*hypD*), which lacks a genomic copy of *hypD* [24]. The plasmids used included: *hypD* alone encoding HypD with a *N*-terminal StrepII-tag (here, referred to as phypD); *hypD* co-expressed with the *hypC* gene, which encodes native HypC bearing a *C*-terminal StrepII-tag (phypDC, [13]); or *hypD* co-expressed with the *hypE, hypF*, and *hybG* genes, the latter encoding HybG with a *C*-terminal StrepII-tag (phypDEFhybG, [25]); note that HypD encoded on phypCD and phypDEFhybG did not carry a tag. After anaerobic growth of the wild-type strain, MC4100, strains DHP-D (Δ*hypD*), and DHP-D transformed with one of the different plasmids, a cell-free extract from each strain was prepared and the total H_2_: BV oxidoreductase enzyme activity present in each extract was determined (Fig. 2a). The results clearly show that, while the extract derived from strain MC4100 had an activity of ∼2.2 units mg^−1^ (Fig. 2a), the extract derived from DHP-D (Δ*hypD*) lacked hydrogenase enzyme activity. Re-introduction of the native *hypD* gene on plasmid phypD restored only approximately 40% of the hydrogenase activity to strain DHP-D, when compared with the activity in the MC4100 extract, while co-expression of wild type (WT) *hypD* with either *hypC*, or with *hypEF-hybG*, resulted in recovery of ∼50% of the hydrogenase enzyme activity in cell-free extracts (Fig. 2a). In contrast, cell-free extracts derived from strain DHP-D transformed with plasmids encoding the HypD_D98A_ amino acid-exchange variant had no detectable hydrogenase enzyme activity (Fig. 2a). A cell-free extract derived from DHP-D transformed with plasmid phypD_S356A_ had a total hydrogenase enzyme activity equivalent to roughly 25% of that measured in the extract derived from MC4100 (Fig. 2a), indicating that this HypD variant retained at least partial functionality in facilitating hydrogenase maturation.

To determine whether the HypD variants were synthesized at similar levels and were stably maintained in the extracts of the strains, an immunoblot using antiserum raised against HypD was performed (Fig. 2b). The results show that wild-type HypD and the HypD_D98A_ and HypD_S356A_ variants were stably synthesized in strain DHP-D and in similar amounts compared with the native HypD protein synthesized from plasmid phypD. Extracts derived from anaerobically-grown strain DHP-D (Δ*hypD*) produced no HypD (Fig. 2b), as expected [24].

Together, these results indicate that the aspartate residue at position 98 in HypD is essential for manifestation of H_2_: BV oxidoreductase activity in *E. coli* and suggest that a lack of, or defective, maturation of the hydrogenases might be the reason for this lack of enzyme activity.

### Synthesis of active forms of Hyd-1, Hyd-2, and Hyd-3 requires D98 in HypD

**Figure 3.**
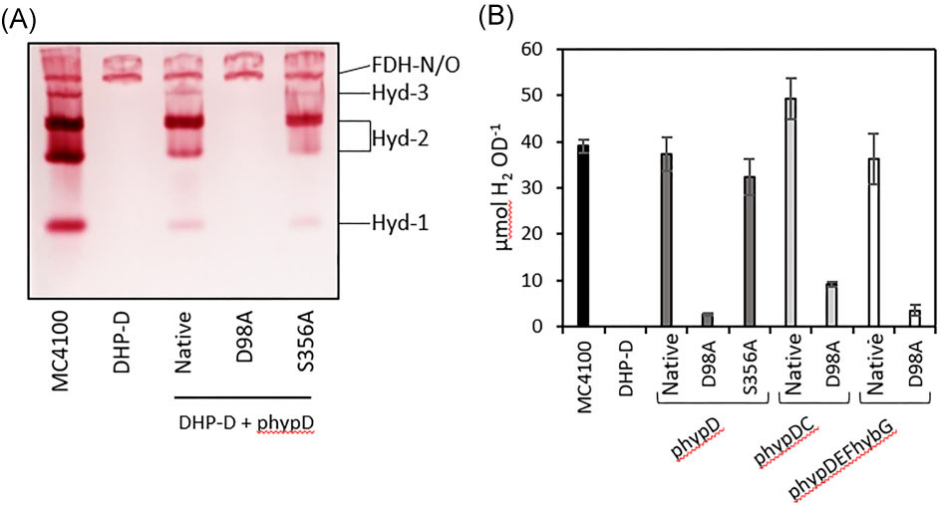
An *E. coli* strain synthesising HypD_D98A_ lacks hydrogenase-dependent H_2_: BV oxidoreductase enzyme activity, but retains low-level H_2_ production. (a) In-gel hydrogenase enzyme activity staining determined in extracts of the indicated strains. aliquots (25 μg of protein) of cell-free extracts were separated in clear-native polyacrylamide (7.5% w/v) gels. See legend to Fig. 2 for strain depictions. A representative activity gel is shown and the experiment was done twice. (b) Aliquots of the headspace from the same strains shown as in part (a) and grown anaerobically for 16 h at 37°C in TGYEP medium (see Experimental) were analysed by GC and accumulated H_2_ was determined. Measurement of H_2_ accumulation was performed with three biological and three technical replicates and the data are presented together with standard deviation.

The total hydrogenase enzyme activity measured using H_2_-dependent reduction of the redox dye BV [26] represents the sum of all three [NiFe]-hydrogenases, Hyd-1, Hyd-2, and Hyd-3, which are synthesized during fermentative growth conditions [14]. The three enzymes can be distinguished, however, after separation of the respective complexes in clear-native-PAGE followed by activity-staining using H_2_, BV, and the redox dye, 2,3,5-triphenyl tetrazolium chloride, which forms a red, insoluble formazan precipitate upon reduction [26, 27]. Analysis of the extracts prepared from each strain using this method clearly showed that all three enzymes can be identified after separation of protein complexes present in extracts of the wild-type strain, MC4100, with Hyd-2 characteristically migrating as two isoforms [26] (Fig. 3a). The extract derived from DHP-D (Δ*hypD*) lacked all three enzyme activities, as expected [24]. Introduction of plasmid phypD, encoding native HypD, into strain DHP-D restored activity of all three hydrogenase enzymes, albeit the activity of each was qualitatively lower than in extracts of MC4100, particularly that of the fast-migrating Hyd-1 (Fig. 3a). The clearly visible H_2_-oxidizing activity of the respiratory formate dehydrogenases, FDH-N and FDH-O, a side activity of these enzymes [28], is independent of the Hyp-protein maturation machinery and served as a useful control showing protein-loading equivalence (Fig. 3a).

Introduction of phypD_D98A_, encoding the HypD_D98A_ variant, failed to restore detectable activity to any of the hydrogenase enzymes, while introduction of plasmid phypD_S356A_ into DHP-D restored activity to all three enzymes and to a level similar to that restored by plasmid phypD encoding native HypD (Fig. 3a). Therefore, the mutation introduced in codon 98 of the *hypD* gene negatively affected the H_2_-oxidising, dye-reducing activity of all three hydrogenase enzymes, while the mutation introduced in codon 356 exerted no negative influence on this activity.

As Hyd-3 is a component of the FHL-1 complex and its proton-reducing activity can be specifically determined without the use of artificial redox dyes, we determined the total amount of H_2_ gas accumulated after 16 h of anaerobic growth at 37°C (Fig. 3b). The results revealed that transformation of strain DHP-D with phypD_D98A_ resulted in a very minor level of FHL-1-dependent H_2_ production, reflecting low-level Hyd-3 activity [15]. Approximately 4% of the level of H_2_ accumulated after growth compared with the amount of H_2_ that accumulated when strain DHP-D was transformed with phypD (Fig. 3b). Introduction of phypD_S356A_ into DHP-D led to an accumulated level of H_2_ gas that was only slightly lower than that observed for the wild-type strain MC4100.

To test whether co-overexpression of the *hypC* gene with *hypD_D98A_* could increase the amount of H_2_ generated by DHP-D, we introduced plasmid phypD_D98A_hypC into DHP-D and this resulted in a two- to four-fold increase in accumulated H_2_ compared with when *hypD_D98A_* was expressed alone from phypD_D98A_ (Fig. 3b). Co-expression of *hypD_D98A_* together with the *hypEF* genes, and the *hybG* gene encoding the Hyd-2-specific maturase, HybG, did not result in an increase in the amount of H_2_ accumulated compared with when the *hypD_D98A_* gene was expressed alone from phypD_D98A_. These data indicate that a low level of Hyd-3 enzyme was generated, despite the D98A residue-exchange in HypD and suggests some limited maturation capability for Hyd-3 was retained by the variant.

### HypC released from its complex with HypD_D98A_ has a barely detectable level of the Fe(CN)_2_CO modification

**Figure 4.**
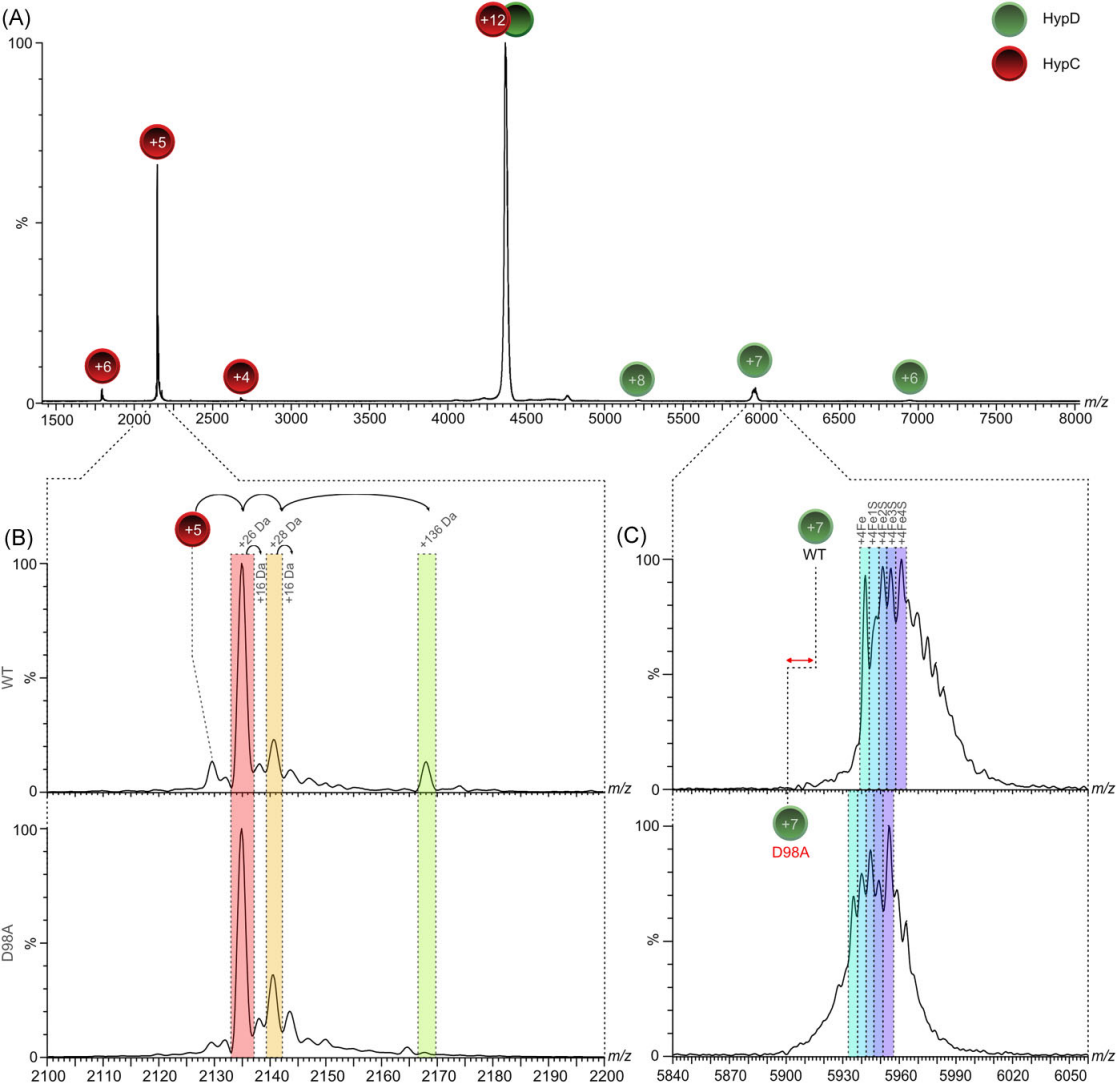
Native MS analysis of native (WT) and mutant HypCD complexes isolated from strain DHP-D. (a) MS/MS spectrum of the dissociation (collision energy: 90 V) of the +12 charge-state of the HypCD complex (1:1) purified from strain BL21 (DE3) transformed with phypDC or phypD_D98A_C. Signals representing separated HypC and HypD are labelled with their corresponding charge states. (b) Inset showing the HypC + 5 charge state originating from dissociation of the +12 charged species of the HypCD heterodimer (part a) at a collision energy of 90 V. Mass shifts of 16, 26, 28, and 136 Da are indicated. The spectrum of HypC released from the native HypCD complex is shown in the upper panel and is referred to as WT on the *Y*-axis, while the spectrum shown in the lower panel is of HypC released from the HypCD_D98A_ complex and is labelled D98A. (c) Inset showing the spectra of WT HypD (upper panel) and HypD_D98A_ (lower panel) +7 charge state originating from dissociation of the +12 charged species of the HypCD heterodimer (part a). Mass shifts due to the presence of the [4Fe-4S] cluster on HypD are indicated in different shading. The *m/z* difference between native HypD and HypD_D98A_ (double-headed arrow) is due to the 44 Da mass-shift between the proteins.

Recent studies using native MS have identified a specific +136 Da modification associated with HypC, and its paralogue HybG, that correlates with the presence of the Fe(CN)_2_CO moiety on the complex [29, 30]. This modification is only clearly observed when HypC is separated from its complex with HypD during native MS (see Fig. 4a and b). Native MS–MS analysis of HypC dissociated from the purified HypD_D98A_-HypC-Strep complex (Fig. 4b, lower panel) revealed that the +136 Da (highlighted in green) modification was only barely detectable after its collision-induced release from the complex, compared to when the same experiment was performed with native HypD-HypC-Strep complexes (Fig. 4b, upper panel). The other so far uncharacterized modifications of +26 Da (red) and +28 Da (orange) observed in the spectra were of similar intensities for both HypC samples (Fig. 4b). The +26 Da modification has been proposed to represent a thiazolidine modification of the *N*-terminal cysteine residue, while the other peak possibly represents bound CO_2_ (addition of 26 + 28 Da) [see 29, 30], although these assignments remain speculative.

### HypD_D98A_ shows a reduced ability to interact with HypE

The accumulated data are consistent with the HypD_D98A_ variant having a severe deficiency in maturation capability and strongly suggest that the protein is impaired in biosynthesis of the Fe(CN)_2_CO moiety. To exclude that this deficiency in HypD_D98A_ function was caused by an improper assembly of the [4Fe-4S]-cluster in the protein, we analysed the integrity of the cluster using native MS [30]. The MS/MS profile of the cluster in HypD_D98A_ and that in native HypD exhibited similar properties and peak intensities (Fig. 4c), but with a mass-shift due to the D98A conversion in HypD_D98A_. These data are consistent with a full-occupancy the [4Fe-4S] cluster in both HypD proteins, and with the intense brown colour of the HypD_D98A_-HypC-Strep complex upon anaerobic purification (data not shown). Moreover, the similar stability of both proteins in cell-free extracts (Fig. 2b) is also consistent with the stable assembly of the [4Fe-4S]-cluster in HypD_D98A_ [23].

**Figure 5.**
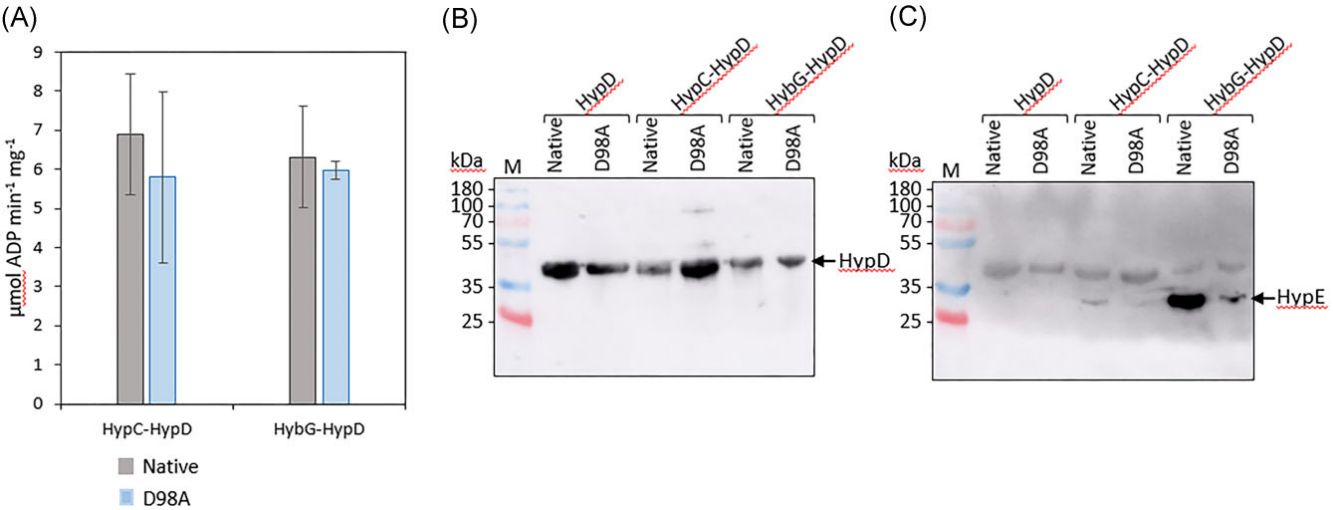
Purified StrepII-tagged HypCD_D98A_ and HybG–HypD_D98A_ complexes show near-native ATPase activity but severely impaired interaction with HypE. (a) ATPase activities of isolated HypCD and HybG–HypD complexes including either native HypD or the HypD_D98A_ variant are shown. Measurement of ATPase activity was performed using three biological replicates, each with three technical replicates and the data are presented with standard deviation. (b) Immunoblot analysis of isolated StrepII-tagged HypD, StrepII-tagged HypCD and StrepII-tagged HybG-HypD complexes (5 μg protein) after SDS-PAGE (12.5% w/v polyacrylamide), including either native HypD or HypD_D98A_, with anti-HypD-antiserum (dilution 1:1000 v/v). (c) Shown is a gel similar to that shown in part (b), originally challenged with anti-HypD antiserum, stripped and then challenged with anti-HypE antiserum (diluted 1:1000 v/v). Molecular mass markers in kDa are shown on the left of each panel. Arrows identify the migration positions of HypD or HypE. the experiments shown in parts b and c were done twice.

Analysis of the ATPase activity [31] of the HypD_D98A_-HypC-Strep and HypD_D98A_-HybG-Strep complexes revealed that they were similar to those determined for the cognate native complexes (Fig. 5a). The ATPase activity of the scaffold complexes is proposed to be involved in the biosynthesis [31], or subsequent transfer [4], of the Fe(CN)_2_CO moiety to the apo-catalytic subunit of the hydrogenase. These findings indicate that a negative impact of the D98A residue-exchange on the scaffold complex’s ATPase activity can be excluded.

As HypE consistently co-purifies in sub-stoichiometric amounts with the HypCD complex [13], which itself has a 1:1 stoichiometry [3, 13, 29, 30], we examined by immunoblotting using anti-HypE antiserum whether HypE was associated with purified HypCD and HybG-HypD native and variant complexes. As a control, native, Strep-tagged HypD and Strep-tagged HypD_D98A_ were also isolated in the absence of either co-overproduced HypC or HypG. Immunoblotting with anti-HypD antiserum revealed that HypD was present in broadly similar amounts in each complex (Fig. 5b). In contrast, anti-HypE antiserum failed to detect any HypE associated with either HypD or HypD_D98A_ when it was not co-overproduced with HypC (Fig. 5c, left lanes), suggesting HypC or HybG is important for stabilizing the interaction with HypE [3].

Analysis of the complexes isolated in association with Strep-tagged HypC detected low, but clearly visible, levels of HypE associated with the native HypCD complex, but consistently lower levels of HypE were associated with the HypD_D98A_-HypC complex (Fig. 5c). However, while co-expression on a plasmid of the native and mutated *hypD* genes with the *hypEF* and *hybG* genes resulted in an intense signal for HypE associated with the native HypD–HybG complex (Fig. 5c), analysis of the purified HypD_D98A_–HybG complex revealed that the amount of HypE associated with the complex was barely detectable (Fig. 5c). These data are consistent with a significantly weaker interaction between HypE and the HypD_D98A_–HybG and HypCD_D98A_ complexes compared with complexes containing native HypD.

## Discussion

Collectively, the evidence presented in this paper suggests that the highly conserved D98 residue in *E. coli* HypD is essential for the generation of active H_2_-oxidising Hyd-1 and Hyd-2 enzymes. When the gene encoding HypD_D98A_ is over-expressed along with *hypC*, then low-level synthesis of active Hyd-3 is retained, which is sufficient to allow a weak FHL-1 complex-dependent H_2_ accumulation, at roughly 10% of the level observed for the wild-type strain. Hyd-3 is synthesized preferentially over the H_2_-oxidizing enzymes [32] and, together with the efficient HypC-dependent maturation of the Hyd-3 large subunit, HycE [21], this presumably accounts for the residual level of synthesis of active FHL-1 complex. This also correlates with the weak, but still detectable interaction between HypE and the HypCD_D98A_ scaffold complex observed in immunoblots (see Fig. 5c) and with the weak signal correlating with the Fe(CN)_2_CO moiety associated with the HypCD_D98A_ scaffold complex analysed by native MS (Fig. 4b).

**Figure 6.**
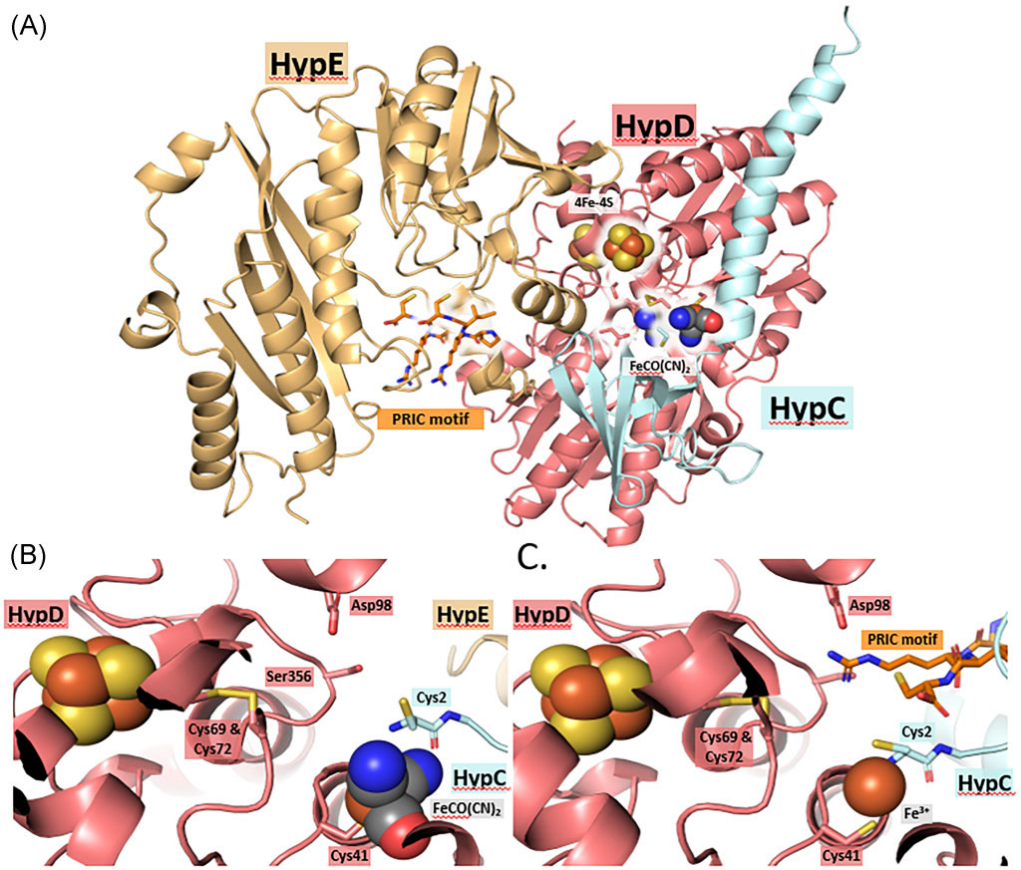
AlphaFold3 predictions for the *E. coli* HypCDE complex and the HypCD complex with the *C*-terminal PRIC-tetrapeptide of HypE. (a) Result of the AlphaFold3 simulation of full-length *E. coli* HypCDE showing the [4Fe-4S] cluster as spheres and the predicted location of the Fe(CN)_2_CO moiety. The key amino acid residues C2 of HypC, the C41, C69, C72, D98, and S346 of HypD, and the PRIC motif at the *C*-terminus of HypE are highlighted in ball-and-stick format. A close-up view around the Fe(CN)_2_CO moiety is shown in b. This prediction has an ipTM score of 0.8 and a pTM score of 0.82. (c) Structure prediction for full-length *E. coli* HypCD together with the *C*-terminal PRIC-tetrapeptide of HypE. The position of the [4Fe-4S] cluster is shown and the predicted location of a Fe^3+^ ion represented as a sphere is also modelled. The prediction has ipTM and pTM scores of 0.88 and 0.91, respectively.

HypE has been shown to co-purify with the HypCD and HybG-HypD scaffold complexes [13]; however, it is typically present in sub-stoichiometric amounts, while HypF is seldom found associated with this complex. The crystal structure of the HypCDE complex from *T. kodakarensis* [20] reveals that the poorly resolved *C*-terminal tail of HypE is localized in proximity to the central cleft in HypD, which includes residue D98. The inability to resolve the *C*-terminus of HypE in the crystal structure [3], which includes the highly conserved PRIC motif shown to be required to deliver the cyano-moiety to the HypCD complex [11, 33], suggests high flexibility of this *C*-terminal tail [20]. It is conceivable, however, that an electrostatic interaction between D98 of HypD and the positively-charged R334 residue of HypE’s *C*-terminal PRIC motif is required to position the thiocyanate moiety on the *C*-terminal cysteine residue of HypE close to the Fe ion proposed to be bound between C41 on HypD and C2 on HypC, thus facilitating the subsequent reductive transfer of the cyanide group to the Fe ion [3, 11, 20]. An AlphaFold3 prediction of the structure of the complex between HypC, HypD and full-length HypE of *E. coli*, together with a fully assembled Fe(CN)_2_CO moiety (Fig. 6a), provides further support for the proposed flexibility of the *C*-terminal tail of HypE; however, all structural predictions that included full-length HypE placed the immediate *C*-terminus of HypE at a site distal to the Fe(CN)_2_CO group (Fig. 6a, b), likely representing the ‘inward’ conformation of the tail, rather than the ‘outward’ conformation proposed to be induced during interaction of HypE with HypCD [3]. However, this may also reflect a modelling bias for unstructured regions. Repeating the AlphaFold3 prediction with HypCD together with only the conserved *C*-terminal PRIC tetrapeptide of HypE’s *C*-terminal tail (Fig. 6c) reveals that the side-chain of R334 is indeed predicted to be positioned within electrostatic distance of the negatively charged carboxylate side-chain of D98. While still speculative, this analysis provides an initial plausible explanation why D98 might be critical for the assembly of Fe(CN)_2_CO group. Moreover, the AlphaFold3 algorithm positioned the PRIC tetrapeptide in the same location within the HypCD complex in 5 out of 5 simulations using the chosen AlphaFold3 settings (see Experimental). Future studies employing cryo-electron microscopy, molecular dynamic simulations or chemical cross-linking might provide further evidence in support of this proposal. Together, our findings are consistent with residue D98 of HypD functioning to stabilize and position the *C*-terminal cysteine residue of HypE close to the bound Fe ion coordinated by C41 of HypD and C2 of HypC to facilitate transfer of the cyanide group [3, 11, 12].

## Experimental

### Bacterial strains used and plasmids constructed

Three *E. coli* K-12 strains were used in this study and included XL-1 Blue {*recA1 endA1 gyrA96 thi-1 hsdR17 supE44 relA1 lac* [F’ *proAB lacIqZ*ΔM15 Tn*10* (Tet^R^)]} (Agilent Technologies Deutschland, GmbH, Waldbronn, Germany), which was used for standard cloning [34], MC4100 (F^−^, *araD139*, ∆(*argF-lac*)*U169*, λ^−^, *rpsL150, relA1, deoC1, flhD5301*, ∆(*fruK-yeiR*)*725*(*fruA25*), *rbsR22*, ∆(*fimB-fimE*) [35], and its isogenic derivative, DHP-D (Δ*hypD*) [24]. The strain used for protein overproduction was BL21(DE3) (Thermo Fisher Scientific, Bremen, Germany).

The plasmids used as templates for mutagenesis were pIBA5-hypD, pT-hypDCStrep [13], and pT-hypDEF-hybGStrep [25], and are referred to as phypD, phypDC and phypDEF-hybG in the current study; only phypD encodes a *N*-terminally Strep-tagged HypD to facilitate enzyme isolation [13]. Introduction of the mutations in codons 98 or 356 in the *hypD* gene of each template plasmid was achieved using site-directed mutagenesis (Q5 Site-Directed Mutagenesis Kit, New England Biolabs) with the oligonucleotides HypDD98A_fwd_ (5′- TACCTTTGGCGCCGCCATGCGCG-3′) and HypDD98A_rev_ (5′- CAGAAGATGACTTCCGGATGGCTGG-3′) and HypDS356A_fwd_ (5′- GATGGTTTCCGCCGAAGGAGCGTG-3′) and HypDS356A_rev_ (5′- AGCGCACCAAACGCGGTT-3′), delivering plasmids phypD_D98A_, phypD_S356A_, phypD_D98A_hypC, and phypD_D98A_hypEF-hybG.

### Growth conditions used

Strains were grown on LB-agar plates or in LB-broth at either 30°C or 37°C [36] for routine cloning experiments. Anaerobic growth of strains for determination of hydrogenase enzyme activity, in-gel hydrogenase activity-staining after native polyacrylamide gel electrophoresis (native PAGE), or for western blotting experiments, was performed at 37°C as standing liquid cultures in the buffered rich medium TGYEP (1% w/v tryptone, 0.5% w/v yeast extract, 0.8% w/v glucose, 100 mM potassium phosphate, pH 6.5) exactly as described [37]. For overproduction of native StrepII-tagged HypC-HypD and complexes of their amino acid-exchange variants, plasmids were introduced into DHP-D (Δ*hypD*) and anaerobic cultivation was in modified TB medium (2.4% w/v yeast extract, 1.2% w/v peptone from casein, 0.04% w/v glycerol, 0.4% w/v glucose, and 0.003% w/v magnesium sulfate heptahydrate) [38]. Strains were grown at 30°C and the growth medium contained 100 μg ml^−1^ of ampicillin to select for plasmid maintenance. Cells were harvested by centrifugation of the culture for 15 min at 50 000 x *g* and at 4°C, and washed cell pellets were either used immediately or flash-frozen in liquid N_2_ and stored at −20°C until use.

### Purification of Strep-HypD and HypDCStrep and HypD-HybGStrep complexes

The *N*-terminally Strep-tagged HypD, and the HypDC-Strep and HypD-HybG-Strep protein complexes (both with a *C*-terminal tag), including those carrying the D98A amino acid exchange in HypD, were purified from BL21(DE3) transformed with plasmids phypD, phypDC or phypDEF-hybG, respectively. All steps including cell growth, cell disruption and protein purification were carried out under anoxic conditions in an anaerobic chamber (Coy Laboratories, Grass Lake, MI, USA), exactly as described [37]. Protein concentration was determined as described [39].

### Measurement of H_2_ production, total hydrogenase enzyme activity and ATPase activity of HypCD complexes

Accumulated H_2_ gas in the gas phase of Hungate tubes after anaerobic cultivation of strains in TGYEP medium for 16 h was performed exactly as described [40]. Hydrogenase enzyme activity in cell-free extracts was determined exactly as described by Ballantine and Boxer [26] and one unit representing 1 μmol H_2_ oxidized min^−1^ mg^−1^ protein, while the ATPase activity of HypCD complexes was determined exactly as described in [31]. Measurements of enzyme activity were performed with minimally three biological and three technical replicates.

### Non-denaturing PAGE and hydrogenase activity staining

Non-denaturing PAGE (polyacrylamide gel electrophoresis) was performed according to [27], whereby aliquots (25 μg of protein) of crude cell extracts, pre-treated for 15 min with 4% (v/v) Triton X-100 at 4°C, were separated in gels, including 7.5% (w/v) polyacrylamide and 0.1% (w/v) Triton X-100. H_2_-oxidizing activity of the hydrogenase enzymes was performed as described [26, 37].

### Denaturing polyacrylamide gel electrophoresis and immunoblotting

Polypeptides in crude cell extracts, or in purified HypCD complexes, were separated by SDS-PAGE (12.5% w/v polyacrylamide), as described [41]. Polypeptides were visualized either by staining with Coomassie Brilliant Blue G250 (Sigma–Aldrich, Germany), or after immunoblotting [42] using antiserum raised against HypD or HypE. After transfer of polypeptides to nitrocellulose membranes, antisera raised against either HypC or HypD were diluted 1:1000 (v/v) prior to use. Full-length gel images were used in this study.

### Native MS of Hyp complexes

Native MS measurements used protein complexes buffer-exchanged into 500 mM ammonium acetate, pH 6.8, as described [29, 30]. The concentration of protein solutions after buffer-exchange was approximately 10 μM. Native MS was carried out on a High-Mass Q-TOF II instrument (Waters Micromass/MS Vision) equipped with a nano-electrospray ionization (ESI) source. Applied capillary voltage was 2.3 kV, with the sample cone voltage set to 160 V. The source pressure was adjusted to 10 mbar and the pressure in the collision cell was adjusted to 10^−2^ to 2*10^−2^ mbar. MS measurements were carried out using MS profile mode for the quadrupole to guide ions within the *m/z* region of interest. Dissociation experiments were carried out by collision-induced dissociation (CID) for the selected ion species. To achieve CID of protein complexes used a collision energy set to 90 V. Data were calibrated by using cesium iodide (CsI).

### Data processing and structural analyses using AlphaFold3

For the AlphaFold3 simulations, Google’s AlphaFold Server was used (https://golgi.sandbox.google.com/) [43]. These simulations were performed on 16th of January, 2025, where CCD codes could be used to add ligands into the simulation, which are normally not offered as an option on this server (SF4 for the [4Fe-4S] cluster and FCO for the Fe(CN)_2_CO cluster). For the interpretation and image generation of the predicted structures, PyMOL 2.3.2 was used.

Amino acid alignments were performed using the Geneious Prime software package (Softwarebox GmbH, Schönaich, Germany).

## Supplementary Material

mfaf014_Supplemental_File

## Data Availability

The data underlying this article are either available in the article or will be shared on reasonable request to the corresponding author.
